# A second puncture and injection technique for treating osteoporotic vertebral compression fractures

**DOI:** 10.1186/s13018-019-1498-x

**Published:** 2019-12-05

**Authors:** Zhaofei Zhang, Feng Jiao, He Huang, Yonghui Feng, Chunliang Xie, Donghua Liu, Fengwei Qin, Sineng Zhang, Peiyu Wu, Weiguang Tan, Wang Tang

**Affiliations:** Department of Orthopedic Surgery, Guangzhou Hospital of Integrated Traditional and Western Medicine, 87 Yingbin Road, Huadu District, Guangzhou, 510800 Guangdong China

**Keywords:** Vertebroplasty, Second puncture and injection, Cement distribution, Osteoporotic vertebral compression fractures, Cement leakage

## Abstract

**Objective:**

To evaluate the clinical effect of the second puncture and injection technique during a percutaneous vertebroplasty (PVP) procedure.

**Methods:**

Patients treated with a second puncture and injection (group A) or a single puncture and injection (group B) during PVP at our institution during 2010–2017 were reviewed. Vertebral height loss, visual analogue scale (VAS) score, Oswestry disability index (ODI), adjacent vertebral fractures, and cement leakage were compared between the groups.

**Results:**

A total of 193 patients were enrolled (86 cases in group A, 107 cases in group B). The follow-up period was 15.64 (12–20) months. The loss of anterior (group A 0.01 ± 0.03; group B 0.14 ± 0.17) and middle (group A 0.13 ± 0.12; group B 0.16 ± 0.11) vertebral height in group B was significantly higher than that in group A (*P* < 0.05). The VAS score and ODI were also significantly higher in group B than in group A at the final follow-up; the VAS score and ODI in group B were 1.65 ± 0.70 and 14.50 ± 4.16, respectively, and those in group A were 1.00 ± 0.74 and 12.81 ± 4.02, respectively (*P* < 0.05). Three patients in group A and two in group B experienced adjacent vertebral fractures. Regarding mild, moderate, and severe cement leakage, there were 25 (29%), 5 (5%), and 0 cases, respectively, in group A and 28 (26%), 3 (2.8%), and 1 (0.009%) case, respectively, in group B (*P* > 0.05).

**Conclusions:**

The second puncture and injection technique may effectively increase the dispersion of cement, thus preventing recompression of the cemented vertebral body, and it does not increase the risk of cement leakage or adjacent vertebral fracture.

## Introduction

Percutaneous vertebroplasty (PVP) has been widely used for treating osteoporotic vertebral compression fractures (OVCFs). PVP can achieve pain relief, reduce bedrest duration, and improve the quality of life in elderly patients. However, with the development of the PVP technique, some related complications have followed, such as recompression of the cemented vertebral body, which can cause a series of problems, such as back pain, limited mobility, kyphosis, neurological compression, and even revision surgery [1–3]. Inadequate cement filling in the vertebral body, especially in the area where the cement is not strengthened between the upper and lower endplates, easily leads to the recompression of the cemented vertebral body [4]. Therefore, how to improve the dispersion of bone cement in the vertebral body during PVP, especially in the unreinforced area between the upper and lower endplates, has become our research direction.

We performed a second puncture and injection technique to improve the dispersion of cement (Mendec Spine, Tecres Medical, Verona, Italy) in these areas that were not strengthened between the upper and lower endplates during PVP (Vertebroplasty System, Guanlong Medical, China). We retrospectively analysed the clinical outcome of patients who underwent this PVP procedure for treating OVCFs and compared the results with the traditional PVP technique (single puncture and injection).

## Materials and methods

### Selection of patients

We retrospectively evaluated a series of patients who underwent PVP between 2010 and 2017 in our hospital. The inclusion criteria were as follows: (1) the second puncture and injection or single puncture and injection techniques was used in a vertebroplasty procedure, (2) patients had a bone mineral density (BMD) below − 2.5, (3) the follow-up was not less than 12 months, (4) a bipedicular approach was used, (5) no symptoms of spinal cord or nerve compression were observed, (6) patients had painful OVCFs with visual analogue scale (VAS) score above 5 points, (7) an ultra-early injection of low-viscosity cement technique was used, and (8) there was a single vertebral body fracture. The exclusion criteria were as follows: (1) patients with angioma or malignancy, (2) patients in whom high-viscosity cement was used, and (3) patients who were lost to follow-up or had a follow-up duration less than 12 months.

The patients who were finally included were divided into two groups according to whether they were subjected to secondary puncture and injection. Those who underwent second puncture and injection were included in the intervention group (group A), and those who underwent single puncture and injection were included in the control group (group B).

### Surgical technique

The patients were placed in the prone position, and the chest and pelvis were elevated with a soft pillow so that there was nothing under the abdomen. The projection of the bilateral pedicles on the body surface was located by C-arm fluoroscopy.

#### Puncture

The puncture needles were delivered to the posterior periosteum of the pedicles after local infiltration anaesthesia with 1.0% lidocaine injection. The needles were advanced forward to point A and continued to enter point B and point C successively, and the needles entered the vertebral body through point C [5].

#### Cement preparation

Sterile water and bone cement were mixed and shocked fully for 1.0 min, after which the combination was loaded into the syringes.

#### Injection of cement

The syringes with cement were attached to the puncture channel and then advanced to the posterior area of the anterior wall of the vertebral body. The cement was injected when the time was 3.0 min. A determination was made whether to continue the injection of cement according to the dispersion of cement in this area. Subsequently, the needles were retracted, and the cement was injected repeatedly. Retraction of the needles was continued until they reached the front of the pedicles, and then cement was injected into this area.

#### Second puncture and injection

**Fig. 1:**
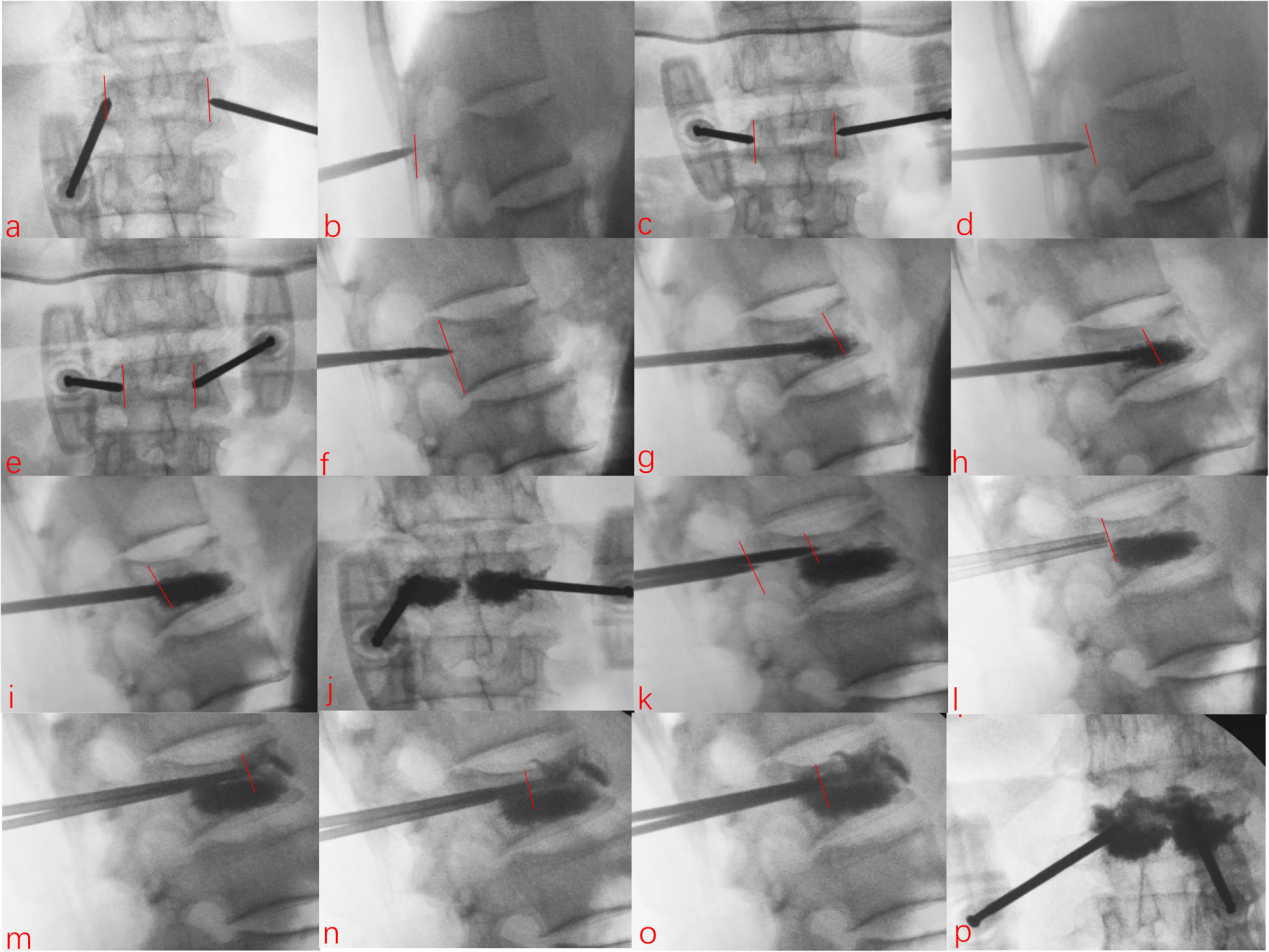
Puncture and injection procedure. **a** The location of point A on the anteroposterior film (the lateral border of the pedicle). **b** The location of point A on the lateral film. **c** The location of point B on the anteroposterior film (the midpoint of the connecting line of point A and the medial border of the pedicle). **d** The location of point B on the lateral film (the midpoint of the connecting line of point A and the posterior margin of vertebral body). **e** The location of point C on the anteroposterior film (the medial border of the pedicle). **f** The location of point C on the lateral film (the intersection of the posterior margin of the vertebral body and the extension line of the connecting line between points A and B). **g** Cement was injected when the needle reached the posterior area of the anterior wall of vertebral body. **h** The needle was retracted and cement injected. **i** The needle was retracted to the front of the pedicles to continue cement injection. **j** Dispersion of bone cement on the anteroposterior film. **k** The needle was retracted to point B, and then, the angle was adjusted to be advanced forward to point C. **l** The needle core was pulled out and prepared to connect the syringes. **m** Same as **g**. **n** Same as **h**. **o** Same as **i**. **p** Dispersion of the bone cement on the anteroposterior film

If the cement was not dispersed below the upper endplate or above the lower endplate according to C-arm fluoroscopy, the needles were retreated to point B immediately, then the puncture angle was adjusted and the needles were advanced forward through point C into the vertebral body. The syringes with cement were connected to the puncture channel, and they were advanced to the posterior area of the anterior wall of the vertebral body (below the upper endplate or above the lower endplate). Subsequently, the cement was injected as above until it reached the front of the pedicles (Fig. 1).

### Parameters observed

Data on the baseline characteristics and surgical parameters of the assessed patients, including sex, age, weight, height, BMD (T score), follow-up time, hospital days, operation time, intraoperative blood loss, and injected cement volume, were collected.

The anterior vertebral height (AVH) and middle vertebral height (MVH), restoration of the anterior vertebral height (RAVH) and middle vertebral height (RMVH), and loss of the anterior vertebral height (LAVH) and middle vertebral height (LMVH) were assessed.

These data were obtained at pre-operation, at 3 days post-operation, and at the final follow-up. For the RAVH, RMVH, LAVH, and LMVH, the values of restoration and loss were respectively defined as the value at the 3-day post-operation evaluation minus the value at pre-operation and the value at the 3-day post-operation evaluation minus the value at the final follow-up. In addition, the VAS score and Oswestry disability index (ODI) values were collected at the three times mentioned above. Cement leakage was also observed and recorded using X-ray or computed tomography (CT) images, and it was divided into mild, moderate, or severe based on Georgy’s classification method [6]. Any adjacent vertebral fracture was assessed by magnetic resonance imaging (MRI) during the follow-up period, and the data were collected.

### Statistical analysis

SPSS 24.0 (IBM Corporation, Armonk, New York, USA) was used to analyse all data.

The continuous data are expressed as the mean ± standard deviation (M ± SD), and the differences between groups were compared by independent samples *t* test. Intragroup differences were evaluated by paired *t* test. The count data were analysed by chi-squared test. *P* < 0.05 was considered statistically significant.

## Results

**Fig. 2:**
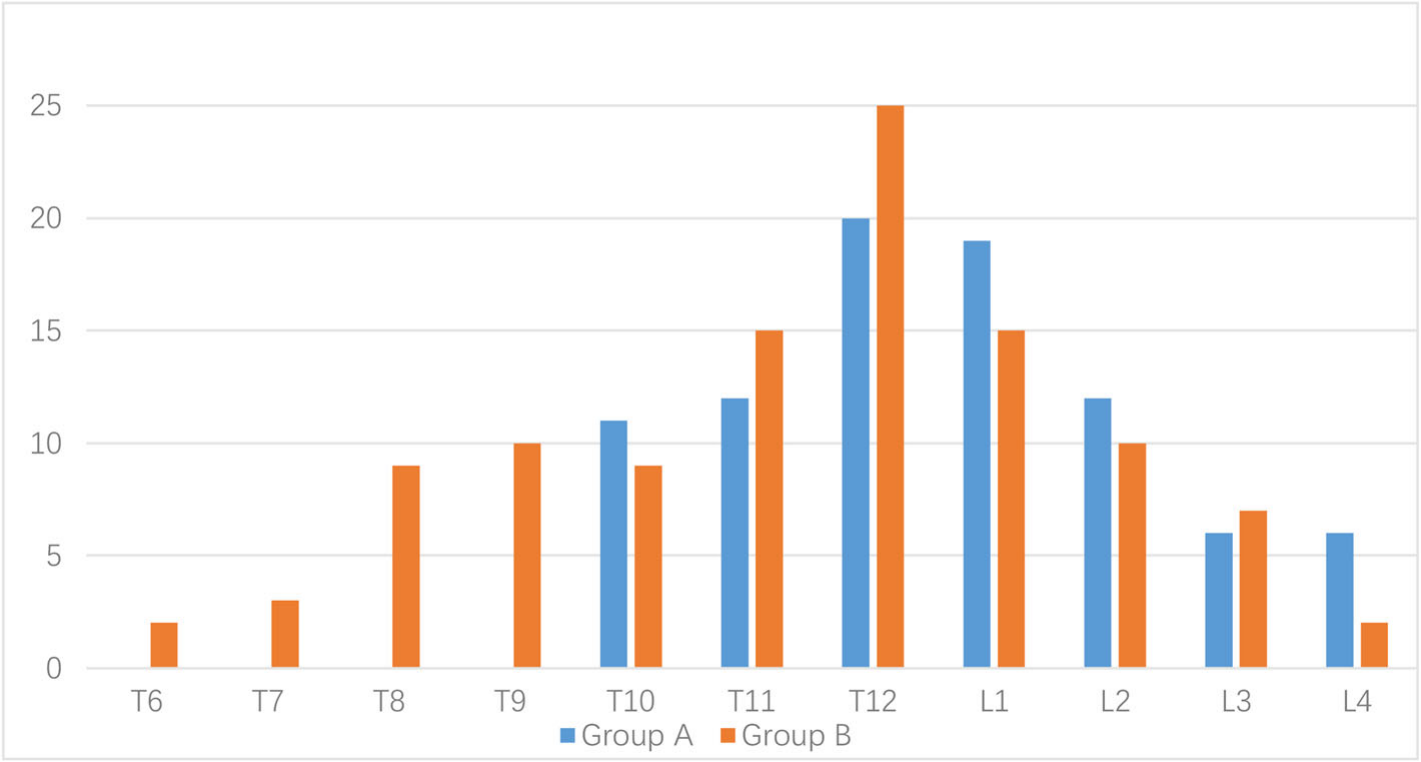
Levels of vertebral fractures

A total of 193 patients with PVP were reviewed, of whom 86 underwent second puncture and injection, and 107 underwent single puncture and injection. We defined these 86 patients and 107 patients as groups A and B, respectively. The final follow-up duration for all patients ranged from 12 to 20 months, with an average of 15.64 months. The cemented vertebral body levels were T_10_ to L_4_ in group A and were T_6_ to L_4_ in group B (Fig. 2). There were no significant differences in sex, age, weight, height, BMD, follow-up time, hospital days, or intraoperative blood loss between the two groups (Tables 1 and 2). The injected cement volume in group A was 2.5–5.9 ml, with an average of 4.10 ml, and that in group B was 1.8–4.3 ml, with an average of 3.24 ml. The operation time in group A (23–55 min; average, 35.49 min) was longer than that in group B (18–61 min; average, 31.30 min) (Table 2).

**Table 1:** Baseline characteristics of the assessed patients

	Group A (*n* = 86)	Group B (*n* = 107)	*P* value
Patients (female/male)	52/34	71/36	0.398
Age (years)	73.51 ± 9.49	72.10 ± 8.02	0.534
Weight (kg)	61.05 ± 9.79	63.49 ± 11.54	0.120
Height (cm)	162.56 ± 8.96	163.12 ± 7.69	0.639
BMD (*T* score)	− 3.64 ± 0.71	− 3.56 ± 0.81	0.475

**Table 2:** Patients’ information

	Group A (*n* = 86)	Group B (*n* = 107)	*P* value
Follow up (months)	15.58 ± 2.57	15.68 ± 2.48	0.783
Hospital days (days)	3.79 ± 1.11	3.71 ± 1.31	0.652
Operation time (min)	35.49 ± 8.96	31.30 ± 9.39	0.002
Intraoperative blood loss (ml)	13.52 ± 2.90	12.66 ± 3.88	0.080
Injected cement volume (ml)	4.10 ± 0.97	3.24 ± 0.75	0.000

AVH and MVH after surgery were significantly restored compared with the pre-operative values in the two groups. Regarding the pre-operative AVH, MVH, RAVH, and RMVH, there were no differences between the two groups (*P* > 0.05). At the final follow-up, the LAVH and LMVH in group A were 0.01 ± 0.03 and 0.02 ± 0.36, respectively, while those in group B were 0.14 ± 0.17 and 0.14 ± 0.13, and the differences were statistically significant (Tables 3 and 4).

**Table 3:** Comparisons of anterior vertebral height at the pre-operation, 3 days post-operation, and final follow-up between the two groups

	Group A (*n* = 86)	Group B (*n* = 107)	*t*	*p*
Pre-operation	2.17 ± 0.23	2.14 ± 0.18	0.933	0.352
3 days post-operation	2.40 ± 0.27^*^	2.44 ± 0.24*	− 0.836	0.404
Final follow-up	2.39 ± 0.27	2.29 ± 0.23	2.587	0.010
Restoration of anterior vertebral height at 3 days post-op	0.23 ± 0.23	0.30 ± 0.21	− 1.891	0.060
Loss of anterior vertebral height at the final follow-up	0.01 ± 0.03	0.14 ± 0.17	7.376	0.000

Restoration of vertebral height: value of 3 days post-operation minus value of pre-operation. Loss of vertebral height: value of 3 days post-operation minus value of final follow-up

*Compared with pre-operation of the same group, *P* < 0.05

**Table 4:** Comparisons of middle vertebral height at the pre-operation, 3 days post-operation, and final follow-up between the two groups

	Group A (*n* = 86)	Group B (*n* = 107)	*t*	*p*
Pre-operation	2.36 ± 0.26	2.38 ± 0.18	− 0.447	0.655
3 days post-operation	2.49 ± 0.26*	2.54 ± 0.20*	− 1.362	0.175
Final follow-up	2.47 ± 0.26	2.40 ± 0.17	2.069	0.040
Restoration of middle vertebral height at the 3 days post-op	0.13 ± 0.12	0.16 ± 0.11	− 1.830	0.069
Loss of middle vertebral height at the final follow-up	0.02 ± 0.36	0.14 ± 0.13	8.525	0.000

Restoration of middle vertebral height: value of 3 days post-operation minus value of pre-operation. Loss of vertebral height: value of 3 days post-operation minus value of final follow-up

*Compared with pre-operation of the same group, *P* < 0.05

Pain was significantly relieved after surgery compared with pre-operative pain in both groups. There were no significant differences in the VAS score or ODI at 3 days post-operation between the two groups (*P* > 0.05). However, the VAS score and ODI in group B were significantly higher than those in group A at the final follow-up; the VAS score and ODI in group B were 1.65 ± 0.70 and 14.50 ± 4.16, respectively, and those in group A were 1.00 ± 0.74 and 12.81 ± 4.02, respectively (Table 5). Three patients in group A and two in group B experienced adjacent vertebral fractures. Regarding mild, moderate, and severe cement leakage, there were 25 (29%), 5 (5%), and 0 cases, respectively, in group A, and 28 (26%), 3 (2.8%), and 1 (0.009%) case, respectively, in group B (*P* > 0.05).

**Table 5:** Comparisons of VAS and ODI at the pre-operation, post-operation, and final follow-up between the two groups

	VAS (scores)				ODI (%)			
	Group A	Group B	*t*	*P*	Group A	Group B	*t*	*P*
Pre-operation	8.52 ± 0.96	8.51 ± 1.05	0.067	0.946	69.88 ± 7.80	70.63 ± 11.64	− 0.535	0.593
3 days post-operation	2.32 ± 0.85	2.46 ± 1.03	− 1.013	0.312	14.55 ± 5.43	15.10 ± 3.96	− 0.778	0.438
Final follow-up	1.00 ± 0.74	1.65 ± 0.70	− 6.216	0.000	12.81 ± 4.02	14.50 ± 4.16	− 2.846	0.005

## Discussion

PVP, as a minimally invasive technique, is an important choice for treating OVCFs. With the development of this technique, however, a series of complications have followed, especially the recompression of cemented vertebral bodies, and it is an important cause of long-term low back pain and kyphosis after PVP [1]. Heo et al. [7] reported that the incidence of re-collapse in 343 patients with OVCFs was 3.21% after PVP. Chen et al. [8] also reported that this incidence was 9.7%, accompanied by post-operative refractory low back pain, limited spinal activity, and other symptoms. What is the cause of vertebral body recompression? Lin et al. [9] retrospectively analysed 137 patients with single-segment PVP and concluded that if the injured vertebral body is not completely augmented with cement, the hard bone cement will destroy the trabecular bone in the unfilled area when an external force acts on the vertebral body, causing these areas to collapse again. Kim et al. [4] revealed a similar result that there is a region that is not cement-augmented between the upper and lower endplates because the bone cement is not sufficiently dispersed, which is more likely to cause recompression of the cemented vertebral body. Liang et al. [10] found through three-dimensional finite element analysis that uneven distribution of bone cement increases the maximum von Mises stress of cancellous bone around bone cement, suggesting that uneven distribution of the bone cement in the vertebral body causes the destruction of the cancellous bone in the unfilled area, leading to recompression of the cemented vertebral body. Zhang et al. [11] also reviewed 177 patients with PVP and found that bone cement distributed around both the upper and lower endplates resulted in a significantly lower incidence of recompression.

These results indicate that the full distribution of bone cement in the vertebral body, especially between the upper and lower endplates, is the key factor in preventing recompression of the cemented body. In our previous study, we applied a technique of accurate puncture and ultra-early injection of low-viscosity cement to improve cement diffusion in the vertebral body [5]. However, some patients still experienced recompression of the cemented vertebral body. We observed that the cement in these re-collapsed vertebral bodies was not sufficiently distributed in the vertebral body, especially below the upper endplate or upon the lower endplate. How to fill these areas with cement during a PVP procedure has become our research focus.

In this study, if an unfilled area, especially upon the lower endplate or below the upper endplate, was found by C-arm fluoroscopy during PVP, we used the second puncture and injection technique. This technique allowed the cement to diffuse into these areas, and we confirmed that the AVH and MVH were not significantly different from those in the control group, and VAS score and ODI were lower than those in the control group at the last follow-up. Most importantly, the incidence of leakage and adjacent vertebral fractures was not increased.

We observed that the post-operative AVH and MVH had different degrees of restoration. We considered that this restoration might be related to the patient’s hyperextended position caused by the patient’s back being elevated with soft pillows before operation, the chest and pelvis being elevated with soft pillows in the prone position during operation, or the pressure from the forward advancement of puncture needles during surgery, rather than related to the PVP procedure itself [12]. This type of restoration will inevitably cause the vertebral body to stretch. If the cement is insufficiently filled in these areas, it is more likely to cause the vertebral body to collapse again [9, 13]. In our study, once these unfilled areas were found during operation, puncture and cement injection were performed again for the unfilled area so that these areas were fully filled with cement, thereby reducing the incidence of recompression. The quality of life of patients was improved, and this was confirmed by the results of the VAS score and ODI. We observed that the VAS score and ODI at the final follow-up in group A were significantly lower than those in group B, indicating that the pain and dysfunction of the patients in group B were more obvious than were those in group A. This result reaffirmed the importance of adequate cement filling to prevent vertebral recompression and improve the quality of life of patients.

This technique of second puncture and injection did not increase cement leakage. We consider this result to be related to the gradual withdrawal of the needles during surgery so that there is space for the cement filling in the front. In addition, the cement is relatively viscous when it is injected again, which may be one of the reasons why it does not easily leak. This type of injection method may cause an increase in the cement volume. Some researchers have reported that an increase in the volume of cement will lead to the occurrence of adjacent vertebral fractures [14]. However, our results are inconsistent with the previously reported result. Lee et al. [15] followed up 351 patients with OVCFs who underwent PVP and found that the average BMD of patients with adjacent vertebral fractures was − 3.1 ± 1.5, while the average BMD of patients without adjacent vertebral fractures was − 2.7 ± 1.5. Therefore, he considered that adjacent vertebral fractures might be mainly due to the natural progression of osteoporosis. Ning et al. [16] also reached similar conclusions by reviewing 365 cases. Consequently, we considered that adjacent vertebral fractures are mainly related to the development of osteoporosis. In addition, this increase in cement volume may play a role in preventing re-collapse. Chen et al. [17] revealed that less bone cement perfusion in the injured vertebral body was an important factor in the loss of vertebral height after surgery. Clark et al. [18] also suggested that adequate perfusion of the bone cement was required for PVP in patients with OVCFs.

This technique of second puncture and injection cannot be performed in all vertebral bodies. In the upper and middle thoracic vertebrae, this technique may present a high risk of neurological or spinal cord injury because the pedicles are relatively narrow. The nearest vertebral body we treated with this technique was T_10_. In addition, it is necessary to carefully measure the size of the pedicles and the angle of the puncture on X-ray and CT images before PVP. This technique is not recommended if the pedicle is too small or the angle of adjustment is too narrow.

We acknowledge that our study has several limitations. First, this is a single-centre study. Second, the sample size is relatively small. Third, there may be some confounding factors or bias because this is a retrospective study. In the next stage, multi-centre, large-sample, and prospective study may be needed for further validation.

## Conclusions

In summary, the second puncture and injection technique can be applied during PVP (below T_10_), especially if the cement is not sufficiently dispersed below the upper endplate or above the lower endplate. This method may effectively increase the dispersion of cement in these areas, thus preventing recompression of the cemented vertebral body; additionally, this technique does not increase the risk of cement leakage or adjacent vertebral fracture.

## Data Availability

There is no any other supporting data.

## Abbreviations

AVH: Anterior vertebral heights
BMD: Bone mineral density
CT: Computed tomography
LAVH: Loss of the anterior vertebral heights
LMVH: Loss of the middle vertebral heights
MRI: Magnetic resonance imaging
MVH: Middle vertebral heights
ODI: Oswestry disability index
OVCFs: Osteoporotic vertebral compression fractures
PVP: Percutaneous vertebroplasty
RAVH: Restoration of the anterior vertebral heights
RMVH: Restoration of the middle vertebral heights
VAS: Visual analogue scale
